# *ABV-CoViD*: An Ensemble Forecasting Model to Predict Availability of Beds and Ventilators for COVID-19 Like Pandemics

**DOI:** 10.1109/ACCESS.2022.3190497

**Published:** 2022-07-13

**Authors:** Vivek Kumar Prasad, Pronaya Bhattacharya, Madhuri Bhavsar, Ashwin Verma, Sudeep Tanwar, Gulshan Sharma, Pitshou N. Bokoro, Ravi Sharma

**Affiliations:** Department of Computer Science and EngineeringInstitute of Technology, Nirma University 56953 Ahmedabad Gujarat 382481 India; Department of Electrical Engineering TechnologyUniversity of Johannesburg 61799 Johannesburg Gauteng 2006 South Africa; Centre for Inter-Disciplinary Research and InnovationUniversity of Petroleum and Energy Studies 199257 Dehradun 248001 India

**Keywords:** Artificial neural networks, ARIMA, COVID-19, healthcare services, IoT, prediction models

## Abstract

Recently, healthcare stakeholders have orchestrated steps to strengthen and curb the COVID-19 wave. There has been a surge in vaccinations to curb the virus wave, but it is crucial to strengthen our healthcare resources to fight COVID-19 and like pandemics. Recent researchers have suggested effective forecasting models for COVID-19 transmission rate, spread, and the number of positive cases, but the focus on healthcare resources to meet the current spread is not discussed. Motivated from the gap, in this paper, we propose a scheme, *ABV-CoViD* (**A**vailibility of **B**eds and **V**entilators for **COVID**-19 patients), that forms an ensemble forecasting model to predict the availability of beds and ventilators (ABV) for the COVID-19 patients. The scheme considers a region-wise demarcation for the allotment of beds and ventilators (BV), termed resources, based on region-wise ABV and COVID-19 positive patients (inside the hospitals occupying the BV resource). We consider an integration of artificial neural network (ANN) and auto-regressive integrated neural network (ARIMA) model to address both the linear and non-linear dependencies. We also consider the effective wave spread of COVID-19 on external patients (not occupying the BV resources) through a $\theta $- ARNN model, which gives us long-term complex dependencies of BV resources in the future time window. We have considered the COVID-19 healthcare dataset on 3 USA regions (Illinois, Michigan, and Indiana) for testing our ensemble forecasting scheme from January 2021 to May 2022. We evaluated our scheme in terms of statistical performance metrics and validated that ensemble methods have higher accuracy. In simulation, for linear modelling, we considered the $ARIMA(1,0,12)$ model, and $N^{8-3-2}$ model for ANN modelling. We considered the $\theta -ARNN$(12,6) forecasting. On a population of 2,93,90,897, the obtained mean absolute error (MAE) on average for 3 regions is 170.5514. The average root means square error (RMSE) of $\theta $-ARNN is 333.18, with an accuracy of 98.876%, which shows the scheme’s efficacy in ABV measurement over conventional and manual resource allocation schemes.

## I. Introduction

In recent times, the world has witnessed the detrimental effects of the novel coronavirus disease-2019 (COVID-19) pandemic. The pandemic spread has been exponential, which has affected communities and masses [1]. The healthcare industry has witnessed stringent bottlenecks during the COVID-19 pandemic, and thus it is critical to leverage smart healthcare to adapt to the rising demands [2], [3]. Recently, in smart communities, Internet-of-Things (IoT) has supported healthcare with effective solutions in medical deliveries, mass surveillance, and contact-tracing mechanisms that have proven pivotal in minimizing the pandemic spread. Despite all the advancements, worldwide, millions of people died due to the unavailability of critical resources to support them. COVID-19 is based on the SARS-CoV-2 virus and majorly causes respiratory problems. Persons suffering from chronic illness, diabetes, and other problems are known to develop critical effects.

**FIGURE 1.**
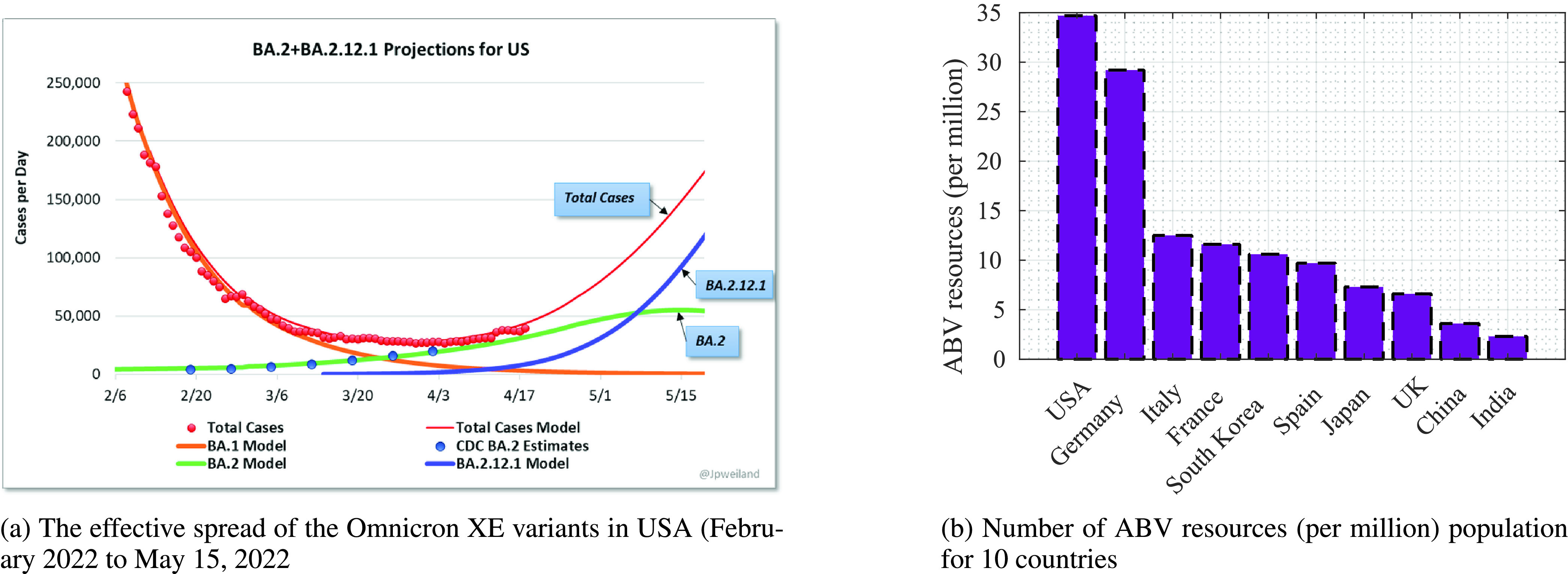
The spread of recent variants of COVID-19, and the BV resource availability for 10 major countries.

On the vaccination front, the study by Oxford Martin School suggests that 65.5% of the world population has received one vaccine dosage, with 11.65 billion doses administered at a global scale [4]. The rate of vaccinations is promising enough, but in reality, many deaths have occurred worldwide. TABLE 1 summarises the total number of confirmed cases and calamities for the top 10 countries, which had the maximum impact on number of COVID-19 cases, and deaths [5]. As per the report by the World Health Organization (WHO), the new Omicron subvariant *BA.2.12.1* and *BA.5* is spreading rapidly in the USA, South Africa, Portugal and India region and is expected to be highly contagious. Another subvariant *BA.2.12.2* is also on the rise, and in combination, they have termed the XE subvariant of Omicron. It is expected to be 25% more contagious than Omicron. FIGURE 1a highlights the number of cases per day (throughout February 2022 to May 2022). A sharp rise is expected after April 2022, and the expected moving rise (indicated in green for *BA.2*) signifies the transmissible effects [6]. Thus, many confirmed cases are expected to hit a peak in USA and India in August 2022. On the other front, the USA has the highest number of critical care intensive units, i.e., beds and ventilators (BV) resource availability, close to 34.7 units per 1 million population. In contrast, India has only 2.3 units per 1 million. FIGURE 1b shows the scenario of the availability of beds and ventilators (ABV) for the 10 countries [7].

**TABLE 1:** Global List of Confirmed COVID-19 Cases and Causalities (as Per WorldoMeter Report on $09^{th}$ May 2022)

Country	Total number of COVID-19 cases	Total number of COVID-19 deaths
USA	8,35,81,715	10,24,546
India	4,31,05,401	5,24,093
Brazil	3,05,64,536	6,64,189
France	2,89,57,421	1,46,724
Germany	2,53,45,357	1,36,925
UK	2,21,14,034	1,76,212
Russia	1,82,27,666	3,76,946
S. Korea	1,75,64,999	23,400
Italy	1,67,98,998	1,64,489
Turkey	1,50,43,379	98,846

As mentioned above, resilient healthcare services are required in operation at massive scales. As COVID-19 mostly affects the respiratory system, resources like beds and ventilators (BV) are extremely crucial [8]. In peak COVID-19 wave condition, many people have suffered due to the non-availability of BVs in hospitals or nearby regions. This also becomes extremely crucial in developing regions, where there is a shortage of hospital services. To fight COVID-19 and future such as pandemics, proper immunization, vaccination, and critical healthcare resource availability are of prime importance.

With potential SARS-CoV-2 virus spread rates, it becomes equally important to analyze the critical healthcare resource availability and further requirements for COVID-19 and non-COVID-19 patients. For COVID-19-positive patients, it is important to isolate them in quarantine zones, hospitals, or at homes, where they are given proper treatment and instruction to recover fast. Recent studies have proposed effective medical delivery and surveillance ecosystems to tackle COVID-19 pandemics. Verma *et al.* [9] proposed a drone-based scheme *VaCoChain*, that is, a vaccination delivery scheme from the production warehouse to nodal centres, and the vaccine registrations are managed in the blockchain. Zuhair *et al.* [10] proposed a UAV-based contact tracing scheme, named as *BloCoV6*, that is for mass surveillance of COVID-19 patients, and the contact tracing records are managed via blockchain. With effective resource orchestration, hospitals are required to set up extensive vaccination and acute-care infrastructures that are set up specifically for COVID-19 facilities. The high dependency unit (HDU) and intensive care unit (ICU) should have a bigger share of beds in hospitals. As a result, ventilators, vital monitors, pulse oximeters, infusion pumps, non-invasive ventilators, and a continual supply of oxygen are in high demand [11], [12]. Aside from treating moderate to severe symptomatic patients, another important consideration is to develop infrastructure to reduce the burden on already overburdened healthcare facilities caused by asymptomatic or mildly infected cases, which will account for nearly 80% of infected cases [13].

Owing to the aforementioned challenges, for fighting the future COVID-19 like pandemics, intensive care unit (ICU) personnel, medical administration, and healthcare stakeholders are required to keep the availability of BV (ABV) resources at designed facility centres so that critically ill patients can be given timely treatment [14], [15]. The only drawback is that the forecasting of COVID-19-positive patients is uncertain. The forecasting models are analysed mainly in cloud servers and require sufficient computational resources for accurate results. This poses serious problems, as it results in over-provisioning or under-provisioning of ABV resources [16]. Another problem is the difficulty in predicting cases during the peak rise. If the forecasting methods work well, it becomes easy to maintain the required ABV resources before the next surge of patients are admitted to the hospitals.

Time-series forecasting models effectively analyze the spread of COVID-19 patients. In time-series forecasting, the observations of a sample variable are used to extrapolate the future values [17]. In linear-based approaches, auto-regressive integrated moving average (ARIMA) models are considered a good fit and are specifically used in stationary time-series models, where it is little or no-missing data [18]. In the ARIMA model, Box and Jenkins [19] presents the auto-regressive (AR) coefficients that are multiplied by past values to get the moving average (MA). However, as a practical limitation, the ARIMA model assumes a linear dependence of the future variable with the current and past variables, which are not always suitable for COVID-19 analysis. Thus, deep learning (DL) based nonlinear models are effectively used in COVID-19 metric predictions. DL models include artificial neural networks (ANNs), long short term memory (LSTM) [20], and autoencoders [21] for analysis on COVID-19 textual and image data.

Recent works suggest that ANNs are suitable for nonlinear forecasting due to their flexible nonlinear function mapping. Also, ANNs have less dependence or assumptions on previous data, making them suitable for COVID-19 adaptive forecasting. ANNs performance also considers multicollinearity, model misspecification, and average smoothing. However, both ANN and ARIMA have their own sets of advantages and drawbacks, and thus a hybrid model is a fit choice. The hybrid models would overcome the limitations of variable selection inaccuracies and improve the overall forecasting accuracy. In hybrid models, a heterogeneous model is more suitable, as it combines the benefits of both linear and nonlinear fit. A popular choice is to fuse ARIMA with the ANN model in a sequential manner, where the ARIMA model is applied to original COVID-19 data, and the residuals are then passed through the ANN model. Another recent study suggests seasonal ARIMA (SARIMA) that includes support vector machines for forecasting [22]. In nonlinear models, apart from ANNs, adaptive time-delay neural networks are also used, mainly in financial stock-market share predictions [23].

However, the forecasting model prediction on the COVID-19 pandemic tends to be inaccurate due to frequent mutation as well as less understanding of the contributing factors of the virus. Thus, genetic knowledge of the virus and its mutation sequences is crucial to boost the model’s accuracy. Thus, an accurate forecast trend of COVID-19 infected patients is uncertain, which makes the BV resource mapping difficult. Moreover, sufficient data points are required for estimations to improve the accuracy. Otherwise, it might generate a biased prediction model [24]. Thus, to effectively model the COVID-19 forecasting, both the short-term and long-term dependency measurements are crucial, as it reports fluctuations, huge confidence intervals, and poor model specifics. Hybridization of linear and non-linear models minimizes time variances and complex auto-correlation structures. A recent study by Chakraborty *et al.* [25] suggests the use of the $\theta $ method, which breaks the original data into $\theta $ lines (curvature in original time-series data, and computes the second-order differences to pass to the autoregressive neural network (ARNN). The ARNN model modifies the ANN models with pre-specified lagged values and fixed neurons in the architecture.

Collectively, with $\theta $-ARNN and ANN-ARIMA models, metric selection to present the forecasting trend is important. Two important selectors, the mean absolute percentage error (MAPE) and directional statistics (DS) should be considered. DS metric considers the accuracy of directional forecasts, which is an important indicator for stakeholders to critically analyse the BV units required at hospitals. In general, DS (sometimes known as circular statistics or spherical statistics) deals with directions (unit vectors in Euclidean space, $R^{n}$), axes (lines through the origin in $R^{n}$), and rotations in $R^{n}$. It is concerned with the observations on compact Riemannian manifolds, such as the Stiefel manifold. With DS, MAPE is important to measure forecast accuracy. It is the most common measurement unit (in %) and is best for data with no extremes.

### A. Design Reasons

**FIGURE 2.**
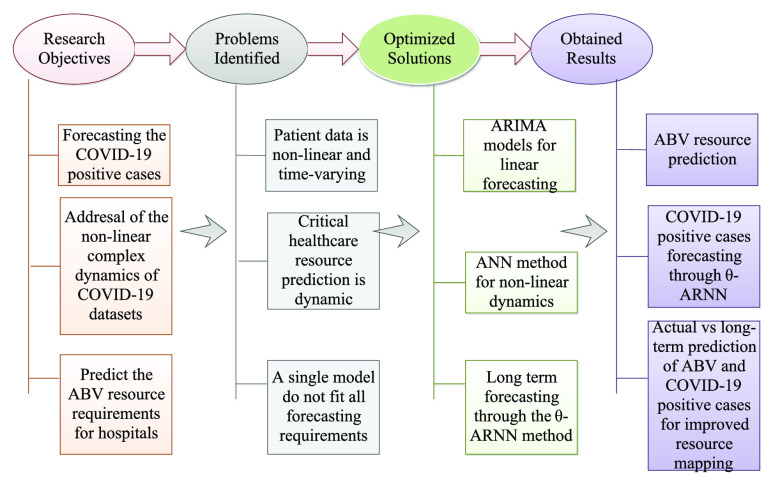
*ABV-CoViD*: Design reasons.

We model the COVID-19 forecasting to predict the ABV resource model. FIGURE 2 presents the design reasons of the proposed scheme *ABV-CoViD*. The collected healthcare data is collected from wireless body area networks (WBAN), and IoT medical setups (sensors), and are communicated to the cloud databases for analysis. Based on the analysis, we form an optimal prediction analysis of the ABV resources in hospitals. With changing mutation, COVID-19 positive cases might vary, and thus, we model the ABV requirement based on the $\theta $-ARNN approach. This allows the model to capture both the linear and non-linear dynamics with variable correlations in the captured data, which improves the overall accuracy of our ABV forecasting. In metric evaluation, we have considered a mean absolute error (MAE) to find the average values of all absolute errors, and mean square error (MSE) to find the average squares, computed as a difference between the estimator and estimated. We also considered root mean squared error (RMSE), which is the difference between the original and predicted values calculated by squaring the average difference across the data set. Afterwards, we used MAPE and DS to compare the results obtained from the aforementioned performance metrics. The detailed explanations and analysis are present in section V. The proposed solutions and the obtained results demonstrate the scheme’s requirement to effectively monitor and predict the BV requirements in real-time in hospital setups.

### B. Novelty

Recent studies have suggested modelling the linear and non-linear dynamics of COVID-19, and have measured a rise in infection rates, deaths, and positive cases. Authors have also suggested directional measurements to mitigate the fluctuations in prediction models. However, for effective treatment of COVID-19 patients at healthcare centres, ABV requirement analysis is equally important. In line with the design reasons specified in FIGURE 2, to the best of our knowledge, the proposed scheme *ABV-CoViD* is a first of its kind to orchestrate effective ABV resource management based on COVID-19 forecasting. A region-based demarcation of BV resources is considered, where we consider two sets of patients- internal and external patients. For internal patients, we monitor the available resources in real-time by analyzing IoT data that is collected from WBANs. For external patients (not admitted to hospitals), the collected BV resource data is sent to region controllers. We consider an ensemble model based on ANN-ARIMA and $\theta $-ARNN approach, which quantifies the positive patient trend, that measures future BV requirements at regions. The $\theta $-ARNN model forms a non-linear prediction of future COVID-19 patients. The step assures that resources are not over-fitted on the model and are sufficiently available whenever required. The performance is considered in terms of different metrics like MAE, MSE, DS, and MAPE. The presented results effectively classify and measure the forecasting trend which makes it easy for the healthcare stakeholders in advance to prepare BV resources at different hospitals.

### C. Research Contribution

The role of disease prevention and control has become more difficult as the COVID-19 epidemic has spread across the world, and the world is facing immense challenges. In this research article, we propose a scheme that forecasts both the short-term and long-term analysis of ABV requirements. Following are the contributions of the article.

• In the proposed scheme, we present a region-wise analysis of the ABV, where the patient readings are collected from local WBAN-based setups and patient health records. COVID-19 patients inside the hospital are mapped to required BV resources through a short-term ANN-ARIMA forecasting, which is based on the patient’s critical condition. This assures that capacity requirement and management inside hospitals.
• Externally, based on BV requirements in different regions, and the COVID-19 positive cases forecast, we notify the local setups (in a region-wise manner) of the future BV requirements. To manage the same, we present a $\theta $-ARNN scheme that forms the prediction on COVID-19 use case patterns, where the complex linear and non-linear residues form the base of the $\theta $ model. This allows a holistic integration and future requirements for external and internal COVID-19 positive patients.
• The performance evaluation is considered on collected data from the world health organization (WHO) COVID-19 USA region dataset. We considered three regions (Illinois, Michigan, and Indiana), and evaluated metrics-MAE, RMSE, MAPE, and DS on different models- ARIMA, $\theta $, ARNN, ANN-ARIMA, and $\theta $-ARNN. The time window is considered from January 2021 to July 2022. We monitor the prediction of BV requirements, positive cases, cumulative deaths, and the impact of vaccination on the total cases.

### D. Layout

The article is divided into five sections. Section II presents the state-of-the-art schemes for COVID-19 identification, and predictions. Section III presents the system model, and the description of entities and the modules setup. Section IV presents the forecasting module in detail, and presents our proposed *ANN-ARIMA*, and $\theta $-ARNN forecasting schemes. Section V presents the metric selection and performance evaluation of the proposed scheme, and summarizes the key findings. Section VI presents the limitations of the proposed schemes, and suggests future research directions, and finally section VII concludes the article.

## II. State-of-the-Art

The section presents the discussion of different state-of-the-art schemes. It presents a comparative analysis of the proposed scheme with other schemes based on different parameters, namely, (1) Pre-diagnosis risk assessment, (2) Post-diagnosis risk assessment, (3) Patient-level population assessment, (4) Hospital level population assessment, (5) Community responses with stronger public health infrastructure, and (6) Healthcare policy with the proposed scheme. TABLE 2 presents the comparative analysis.

**TABLE 2:** Comparative Analysis of ABV-CoViD With Existing State-of-the-Art Schemes

Authors	Year	1	2	3	4	5	6	Advantage	Limitation
Khashei et al. [26]	2011	✓	✓	X	X	X	X	A hybrid ANN-ARIMA approach where a time-series data is decomposed into linear and non-linear components	Model performance improves in isolation, but in combination the performance degrades, which suggests improvements are required in the selection of tuning hyperparameters
Gao et al. [27]	2019	✓	✓	X	X	X	X	An average mutual information (AMI) based scheme for complex data decomposition.	Improvement is required to handle forecasting intrinsic mode function (IMFs) with high accuracy
Alaa et al. [28]	2020	✓	✓	X	✓	X	X	Patients with personalized management plan	Effective collaboration is a challenging criteria.
Shokoohi et al. [29]	2020	X	✓	✓	X	X	✓	Focus is to strengthen the healthcare system and handle patients data	Failure to consider the golden window opportunity
Xu et al. [30]	2020	✓	✓	X	X	X	X	An IMF based scheme which uses the ensemble empirical mode decomposition with ARIMA and ANN	More parameters for accurate forecasting is to be explored.
Lythgoe & Middleton [31]	2020	X	✓	✓	✓	X	X	Focus on clinical trials and the potential outcome were analysed	Challenge to create the the high quality data that can be used to assess potential therapies for both the treatment and prevention of this global emergency.
Cao et al. [32]	2020	✓	✓	X	X	X	✓	Identification of the contingency plan	Interim management techniques were unable to with stand a large scale out break.
Ali et al. [33]	2020	✓	✓	X	X	X	X	To create awareness among the public to prevent, manage, and treat COVID-19	There is a need for the collective global efforts to fight against future pandemics.
Schaar et al. [28]	2021	✓	✓	✓	✓	X	X	Identification of the clinical decision support system	For guiding hospitalization and treatment decisions, quantifying uncertainty in forecast of patient level outcomes is critical.
Roberts et al. [34]	2021	X	✓	✓	X	✓	X	A systematic review for AI models for COVID-19 CT image diagnosis.	The authors excluded several high-quality papers due to lack of documentation, algorithmic approach, and results reproducibility.
Qiang et al. [35]	2021	X	✓	✓	✓	✓	X	An empirical mode decomposition (EEMD) and ensemble IMF model using ARIMA to forecast COVID-19 outbreaks in Pakistan.	The models are defined to predict short-term strategies, and not focused on long-term implications.
Lakshmi P et al. [36]	2021	✓	✓	X	X	X	X	Identification of the low power consumption operations	Trust factor is the critical challenge
Tabish et al. [37]	2021	✓	✓	X	X	X	✓	Different treatment policies for the different patients	Medical waste, such as contaminated masks and gloves, as well as wasted or expired pharmaceuticals, is a problem.
Gaetano Perone [38]	2022	X	✓	X	X	✓	✓	Both short-term and medium-term prediction is achieved on COVID-19 deaths using the SARIMA model	Prediction beyond 15 to 20 days boundaries might not be accurate, and thus a reliable estimation is not achieved.
Chandra et al. [39]	2022	X	✓	✓	X	X	✓	A multi-step COVID-19 fourth wave forecasting is done using a bidirectional LSTM model	The accuracy might vary due to modelling differences of diverse parameters like population density, cultural lifestyle, and social aspects of the considered population
Allah and Hassanien [40]	2022	✓	✓	✓	X	✓	✓	A forecasting model ISACL-MFNN is proposed that integrates interior search algorithm on chaotic learning in multi feed-forward neural network. The network is trained by specifically considering the local optimal and achieves high accuracy	Critical health resource availability, and mapping are not considered in the study
Jeanne et al. [41]	2022	X	X	✓	✓	✓	X	Determined the impact of specific socioeconomic factors on the number of cases observed globally	Medical solutions must be closely linked to organizational and behavioral solutions
Bhattacharya et al. [42]	2022	✓	✓	✓	X	X	✓	A hybrid forecasting model on five countries USA, Brazil, India, Canada, and UK to predict the daily new COVID-19 using the $\theta $-ARNN method	Spatially heterogeneous uncertainties are not covered in greater detail
Changjia et al. [43]	2022	X	X	✓	✓	✓	X	A dynamic model is simulated that suggest the emergency medical resource based on disease progression and the time duration of the medical resource required	Recommendations are purely based on the trends and progression of COVID-19.
Proposed	2022	✓	✓	✓	✓	✓	✓	Machine learning (ML) approaches are used to predict the requirements of the Beds and ventilators for the patients management	Trust factor is not considered here.

1-Pre-diagnosis risk assessment, 2-Post-diagnosis risk assessment, 3-Patient level population assessment, 4-Hospital level population assessment, 5-Community responses with stronger public health infrastructure, 6- Healthcare policy, ✓ - denotes the parameter is present, X - denotes the parameter is absent.

For COVID-19 response and incident systems, Kashei *et al.* [26] presented a hybrid ANN-ARIMA model for time-series analysis. The model captured the linear dependencies via ARIMA, and non-linear dependencies are captured through the ANN model. Gao *et al.* [27] presented a reconstruction of deterministic forecasting for crude oil price prediction based on average mutual information (AMI), where complex data is decomposed into modes and reconstructed into intrinsic mode functions (IMF) composition. The verification is carried out on the Brent and West Texas Intermediate (WTI) crude oil dataset, and benchmarking is done through ARIMA, generalized autoregressive conditional heteroscedasticity (GARCH) model, and ARIMA Kaman filter model. The authors concluded that the proposed model took fewer resources for price forecasting, due to intrinsic IMF breakup and reconstruction modes. Alaa *et al.* [28] proposed an approach where they focused on the personalized management plans of the patients for their severity level, along with the collaboration between the policy guidelines laid by COVID-19 health precautions and its address in hospital setups. Shokoohi *et al.* [29] strengthened the healthcare systems and handled patient information. Still, the approach did not consider the golden window opportunity, where the resource management is synchronized with the count of COVID-19 positive patients.

Xu *et al.* [30] presented forecasting on crude-oil price prediction based on IMF decomposition and reduced the computational complexity of forecasting. The model is based on the autocorrelation of all IMFs and presented an ensemble empirical mode decomposition (EEMD) approach for the decomposition of all IMFs. The analysis is carried out on the Brent and WTI datasets, and it is found that the proposed scenarios have a lower computational time, with higher accuracy. The study is limited in terms of stochastic detailing of IMFs and the model performance on crude oil prediction. More parameters like the wind speed, and electricity cost needs to be considered as future aspects of the study. Lythgoe and Middleton [31] proposed a system for the clinical trials and its analysis to find the optimal solution to manage the patients in a pandemic situation. The main challenge in the scheme was to consider high-quality data that can be used to assess potential therapies for both the treatment and prevention of this global emergency.

Cao *et al.* [32] addressed the problem of authors in [31], and identified a contingency plan, but the scheme was non-scalable for a global outbreak. Ali *et al.* [33] created an awareness among the public to prevent, manage, and deal with the COVID-19 situation, but their proposed scheme lacked the collective efforts to fight against a mass-scale pandemic. Vander *et al.* [28] proposed a clinical decision support system. The drawback of this system is the uncertainty in forecasting the patients count in complex scenarios. Roberts *et al.* [34] presented a review on the usage of ML approaches to predict computed tomography (CT) image diagnosis, and shows the potential limitations of heterogeneity in gathered images from different hospitals, which limits the accuracy of the models. Qiang *et al.* [35] presented a forecasting trend of COVID-19 infections, deaths, and recoveries in Pakistan country. The data is considered till July 2021, and it is decomposed using the EEMD technique. Once EEMDs are formed, IMFs are computed and the ARIMA model is applied. Thus, an ensemble EEMD-ARIMA model is discussed in the work, and it is predicted that with 95% accuracy the infection rate would increase 1.46 times in the Pakistan population. The paper presented that almost an increase of 1.54 times would be recorded in deaths, and thus the model presents insights to the government to take realistic measures and acquire sufficient resources to handle the pandemic. Lakshmi *et al.* [36] identified an approach to identify the low power consumption operations related to the management of the patient’s information, but trust factors are not discussed. Tabish *et al.* [37] proposed an approach to identify the different treatment techniques and policies for the different patients with different levels of severity.

Researchers are working on different forecasting models to predict expected hospital resources, Gaetano Perone [38] proposed a forecasting model for mid-term cumulative deaths from the pandemic in 12 countries and used an integrated ARIMA and SARIMA model. As a result, the authors obtained 95.8% MAE, which shows a seasonal pattern for the COVID-19 pandemic. The approach can be used to form BV resource predictions in our healthcare setups. However, predictions beyond the 15 to 20 days window are not much accurate, and thus resource predictions beyond these boundaries might not be accurate at massive scales. Chandra *et al.* [39] proposed a recurrent neural network and long short term memory based hybrid model to predict COVID-19 infections [44]. The authors identified the COVID-19 hotspots in India for both in first and second waves. They have predicted the chances of another wave in October and November 2021 is less, which gives an estimate to healthcare authorities about the critical resources in those months. However, the scheme heavily depends on collected data and captured dependencies such as population density, demographics, and lifestyle, which might be inaccurate to generalize for the entire population. Allah and Hassanien [40] proposed a scheme *ISACL-MFNN* presented an improved interior search algorithm that uses a multi-feed forward neural network for COVID-19 positive cases forecasting from 22 January 2022. The network is trained by tuning its parameters to optimal values, which allows the scheme to have high accuracy. The simulations are carried out in three countries: USA, Italy, and Spain. However, the data is mainly taken from open datasets, and real-time clinical data is not considered in the study. Jeanne *et al.* [41] combined economic variables and globally collected infected patients data and drew up a linear regression model to identify the economic factor affected by the number of increasing COVID-19 positive cases worldwide. Bhattacharya *et al.* [42] proposed the $\theta $-ARNN approach as an effective forecasting that captures the linear and non-linear patterns of COVID-19 patients. Authors in [43] have proposed a dynamic model that identifies the key factors in the allocation scheme. The model is simulated in Shanghai city of China, based on the pandemic data of Wuhan city. The model identifies the requirement of the medical resources and based on the requirement, the recommendations are posted to healthcare setups for emergency and response systems.

Based on the aforementioned discussions, our proposed scheme explicitly presents a mapping to the healthcare resource required with the impact of COVID-19 cases that allows the hospital authorities to get a clear picture of BV required at local setups. The scheme collects the BV requirements region-wise and sends them to the cloud servers for analysis on both the linear and non-linear dependencies. We have considered both the short and long-term factors while computing the ABV requirements.

## III. ABV-COVID: System Model and Modules

In this section, we present the details of the proposed scheme *ABV-COVID* that measures the real-time ABV requirements for the COVID-19 affected persons. We start by presenting the scheme’s system model and modular flow as follows. Next, TABLE 3 presents the list of acronyms and mathematical notations used in the paper.

**TABLE 3:** Acronyms and Notations

Acronyms and notations	Descriptions	Acronyms and notations	Descriptions
$\theta (W)$	Degree of polynomial	$R_{total}$	Total availability of the resources
$O_{l}$	Oximeter readings	$T''(\theta)$	Second order differential
$\hat {E}_{t}$	Sum of terms for additive forecast	$T_{l}$	Temperature reading
$\phi (W)$	Degree of polynomial	ABV	Availability of Beds and Ventilators
$a_{\theta }$ and $b_{\theta }$	Trend coefficient	ANN	Artificial Neural Network
$d(P,H)$	Euclidean distance	ARIMA	Autoregressive Integrated Moving Average
$E_{DHO}$	district hospital Organizations	ARIMA-ARNN	ARIMA-Autoregressive neural networks
$E_{H}$	Number of hospitals	ARNN	Autoregressive neural networks
$E_{RC}$	Region controller	BSN	Body Sensor Network
$E_{RGA}$	Region Government Authority	CC	Cloud Computing
$E_{P}$	Potential Patients	COVID-19	Novel Coronavirus Disease 2019
$f$	lagged input	HDU	High Dependency Unit
$g$	hidden layers	ICU	Intensive Care Unit
$H_{k}$	Hospital resources	IoT	Internet of Things
$L_{t}$	Linear component	LPU	Local Processing Unit
$NL_{t}$	non-linear component	MAE	Mean absolute error
$P_{l}$	l patients	ML	Machine Learning
$P_{sink}$	Sink node	RMSE	Root mean square error
$P_{sr}$	Source node	TARRN	$\theta $-ARNN
$Q_{max}$	Hospital’s maximum resources	$(R1,R2, R3)$	Regions

### A. System Model

**FIGURE 3.**
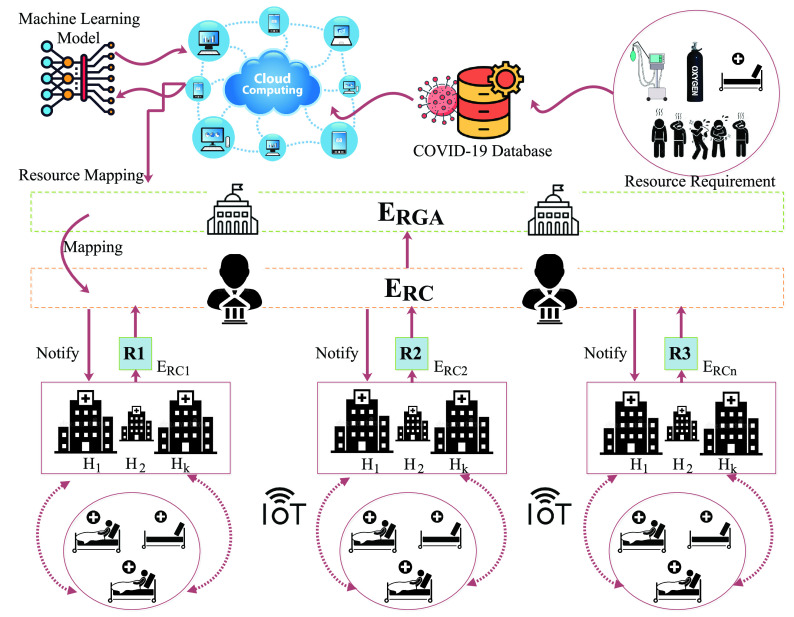
*ABV-COVID*: The system model.

In this section, we present the system model of the proposed scheme. FIGURE 3 presents the details. In the proposed scheme, we consider an entity set $E=\{E_{H}, E_{RC}, E_{RGA}, E_{P}, E_{DHO}\}$, where $E_{H}$ denotes the hospitals, $E_{RC}$ denotes the region controller, $E_{RGA}$ denotes the region government authority, $E_{P}$ denotes the potential patients, and $E_{DHO}$ denotes the district hospital organizations. We consider a state-wide administration in the proposed scheme, which is divided into $n$ regions, depending on local jurisdiction of $E_{RC}$. The regions are denoted as $R=\{R_{1},R_{2}, {\dots },R_{n}\}$, with associated $\{E_{RC_{1}}, E_{RC_{2}}, {\dots }, E_{RC_{n}}\}$. In any particular region $R_{n}$, we assume $k$ hospitals are operational, denoted as $\{H_{1}, H_{2}, {\dots }, H_{k}\}$. In any hospital, we assume there are already $l$ patients, $\{P_{1}, P_{2}, {\dots }, P_{l}\}$. Any $l^{th}$ patient in $H_{k}$ might utilize hospital resources in $H_{k}$ (beds, ventilators, and medical equipments). In the scheme, we confine our discussion to COVID-19 monitoring, and assume that an IoT-enabled sensor ecosystem setup on $E_{l}$. We consider two-sensors units (temperature $T_{l}$, and pulse oximeter $O_{l}$) are used to measure body temperature, and oxygen levels in blood. The collected readings $D_{l}=\{T_{l}, O_{l}\}$ for all $l$ patients are accumulated by $E_{H_{k}}$, and send to a local processing unit (LPU). Based on a specified threshold $T_{c}$, we determine the criticality of $E_{P}$, and hospital resources are assigned accordingly.

At any $H_{k}$, the critical resources $CR=\{BA, V, OC\}$ are considered that denotes the bed availability, ventilators, and oxygen cylinders, respectively. Real-time resource updations are maintained at $\{H_{1}, H_{2}, {\dots }, H_{k}\}$ by $E_{H}$. At any given time $t$, $E_{RC}$ fetches $R~\forall ~H_{k}$ and maintains the data on a private server, which is only accessible by authorized stakeholders. At all $\{R_{1}, R_{2}, {\dots }, R_{n}\}$, $E_{RC}$ fetches database of COVID-19 patients from $E_{RGA}$, that shows the COVID-19 positive patients. The affected patients must be mapped to available resources $CR$ for every region. To ensure the real-time mapping, we run an ML-based analytics model on a private cloud server that involves an ARIMA-based model to analyze the time-series forecasting on available resources at $H_{k}$. The model assigns an $E_{P}$ to a particular $H_{k}$ and, based on criticality, assigns only the required $CR$. Thus, at all times, optimized resource selections are possible. We next discuss the modular flow of the proposed scheme.

### B. Modules Setup

In this subsection, we discuss the flow of the proposed scheme and define the module setup and communication flow. First, we consider a module-wise setup in the scheme, and 4 modules are considered, denoted as $\{M_{1}, M_{2}, M_{3}, M_{4}\}$. $M_{1}$ denotes the Local Hospital Setup, $M_{2}$ denotes the region-wise availability of the hospital resources (vacant beds, ventilators, and oxygen cylinders), $M_{3}$ denotes the region-wise COVID-19 patient database setup, and $M_{4}$ denotes the cloud-based analytics.

#### 1) $M_{1}$-Local Hospital Setup

In this module, we consider the local hospital setups $\{H_{1}, H_{2}, {\dots }, H_{k}\}$. Any $l^{th}$ patient $P_{l}$ is equipped with $\{T_{l}, O_{l}\}$, and other associated sensor units, if required. In the body sensor network (BSN), we consider the source and sink nodes as $P_{sr}$, and $P_{sink}$, respectively. Thus, in the proposed scheme, we constrain $P_{sr}$ to $\{T_{l}, O_{l}\}$, and LPU acts as the sink, where the readings are collected. To determine $T_{c}$, we consider two conditions as follows.

\begin{align*} T_{l}>&99 F \\ O_{l}>&96\%\tag{1}\end{align*}

For any $l^{th}$ patient, we consider these conditions, and if both conditions holds *TRUE*, we move further to his isolation at $H_{k}$. In case any condition is *FALSE*, we keep $P_{l}$ mapped to $H_{k}$. A double-check is presented for non-critical patients. We monitor the $P_{l}$ health condition and compute the days $P_{l}$ has stayed. If the number of stayed days is more than the isolation period, i.e., (14 days), then the patient is considered to be non-critical, and his entry mapping is removed from $H_{k}$. Thus, the critical resource set $CR$ is incremented, which can be assigned to other patients. We consider that at any given time $t$, $q$ patients are admitted at $H_{k}$, and thus the count becomes $(l+q)$. We consider that $s$ BAs, $r$ V, and $t$ OCs are available at time $t$, and $w$ beds are available, where $w>l$. Thus, $(w-l)$ beds can be allocated, where $(w-l)>q$ is considered to avoid bottlenecks. The next module depicts the computation at a larger scale, where we compute the availability of $n$ regions.

#### 2) $M_{2}$-Region-Wise Availability of Hospital Resources

As depicted in $M_{1}$, $E_{RC}$ computes the availability of $CR$ in $H_{k}$. In $M_{2}$, we consider a region $R$ that has $k$ hospitals, and every hospital announces its resource availability, denoted as $\{CR_{1}, CR_{2}, {\dots }, CR_{k}\}$. In general, we have $n$ regions, and thus a mapping function $M_{2}: R \rightarrow CR$ is set up. We consider a notation $CR_{k}^{n}$, that denotes that $k^{th}$ resource is available in $n^{th}$ region. Once resources for all $\{R_{1}, R_{2}, {\dots }, R_{n}\}$ is collected, we denote the total availability as $R_{total}$, which is depicted as follows.

\begin{align*}&R_{total} = \{CR_{1}^{1}, CR_{2}^{1}, {\dots }, CR_{k}^{1}, \tag{2}\\&CR_{1}^{2}, CR_{2}^{2}, {\dots }, CR_{k}^{2}, {\dots }, CR_{k}^{n}\}\tag{3}\end{align*}

Once the $k \times n$ matrix $M$ is evaluated, the details are presented to $E_{RGA}$ from $E_{RC}$. In the matrix, we denote an entry $a_{ij}$ as 1 if resource is available, otherwise $a_{ij}$ is 0.

#### 3) $M_{3}$-COVID-19 Patient Database Setup

In this module, we compute the external resource requirements for potential COVID-19 patients. To formulate, we consider that in any $R_{n}$, there are $t_{n}$ persons. Out of $t_{n}$, we assume that $q_{n}$ patients have tested themselves at COVID-19 centers, and detected as *POSITIVE*. Thus, $E_{RC}$ collects the data for $q_{n}$ patients from $R_{n}$. In totality, we have $\{q_{1}, q_{2}. {\dots }, q_{n}\}$ potential patients (mentioned as $q$ in $M_{1}$), which can be allocated to any $H_{k}$. These $q$ patients data is forwarded by $E_{RC}$ to $E_{RGA}$ that stores the data on a private cloud server for faster processing. An encryption key $K_{RGA}$ is shared with authorized stakeholders to access the cloud. $E_{RGA}$ also collects $R_{total}$, and runs a ARIMA model for moving average prediction for the next $f$ days. The ARIMA model presents the prediction on $q$ moving pattern, and a real-time match availability to $R_{total}$ is computed as output.

#### 4) $M_{4}$-Cloud-Based Analytics

This module runs $E_{RGA}$ the ARIMA model on cloud server $C$ based on two inputs, $R_{total}$ and $q$. We find the prediction of $q$ for the next $f$ days and figure out whether optimal resources in $R_{total}$ to support the cause are available or not. For any given patient $P$, we map to the nearest region $R$ in case of a critical situation. For the same, we compute the real-time location coordinates $(x_{p}, y_{p})$ of $P$, and locate nearest $H_{k}$ in $R$, as the data of regions are available to $E_{RGA}$. A Euclidean distance formulation is considered to find the shortest distance for the patient to the hospital. We consider the set of patient points as $P$, representing the spatial locations. We define a real-valued function $d(P, H)$ to denote the distance from $P$ location to hospital $H_{k}$ location. The distance function is then computed as follows.

\begin{equation*} d(P,H) = |\sqrt {(x_{h}-x_{p})^{2}+(y_{h}-y_{p})^{2}}|\tag{4}\end{equation*}

Thus, this allows a timely action of orchestrating critical health resources to $P$. Once any new patient is admitted in $H_{k}$, a resource $r \in R_{total}$ is allocated to the patient. In the proposed scheme, we will discuss the ARIMA model. Algorithm 1 presents the details of region-wise resource mapping of $CR$ and checks whether any $l^{th}$ patient suffering from COVID-19 is provided treatment at $H_{k}$. Lines 1–6 of the algorithm loop through the hospital and patient sets and maps the patient to a hospital, where $P_{l}$ is equipped with sensors in the WBAN network. Based on required checks, the criticality $C[j]$ for any $j^{th}$ patient. We check the maximum resource $Q_{max}$ and assign the patient the resource at $H_{k}$. At the server, we set up an ANN-ARIMA forecasting model that leverages $E_{RSA}$ to collect $R_{total}$. For linear conditions, $y_{t}$ is generated. In case a patient is not positive, we do not map the required resource sets and flag $C[j]$ as *FALSE*. Lines 7–23 depict the conditions. Lines 24–33 forms the $k \times n$ matrix $M$, the details of which are presented to $E_{RGA}$. For external patients, lines 34–36 collect the data of external COVID-19 positive persons who currently are not allocated any $H_{k}$. We execute the $\theta $-ARNN model to predict the forecast

Algorithm 1 **Resource Mapping of Beds and Ventilators in ABV-CoViD Scheme**
Input: Hospitals $\{H_{1}, H_{2}, {\dots }, H_{k}\}$, patients $\{P_{1}, P_{2}, {\dots }, P_{l}\}$ with equipped sensors $\{T_{l}, O_{l}\}$.
Output: Region-wise requirements of resources to compute $R_{total}$. A boolean flag $B=\{0,1\}$ indicates whether resource is allocated to patient.
1: **for** $i \leftarrow $ 1 to $k$ **do**
2: **for** $j \leftarrow $ 1 to l **do**
3:
$Q \leftarrow $ Map ( $H_{i}, P_{j}$)
4:
$E_{H_{k}} \leftarrow $ *Collect_Sensor_Data* ( $T_{j}, O_{j}$)
5: **if** ( $T[P_{j}] > 99F$ and $O[P_{j}]>96\%$) **then**
6:
$C[j] \leftarrow $ *TRUE*
7: **if** ( $Q < Q_{max}$) **then**
8: Assign( $Q \rightarrow P_{j}$)
9: Store $(C[j], Q)$ at server $R$
10: Call *ANN-ARIMA* ( $p,d,q, R_{total}$) to generate linear $y_{t}$
11: **else**
12: Print *“Mapping of resource not possible”*
13: **end if**
14: **else**
15:
$C[j] \leftarrow $ *FALSE* and output $P_{j}$ as non-critical
16: **end if**
17: **if** ( $C[j]==TRUE$ and $D(P_{l}) > 14$) **then**
18:
$C[j] \leftarrow $ *FALSE*
19: Remove $P_{j}$ from $Q$ list
20:
$Q \leftarrow \,\,Q-1$
21: **end if**
22: **end for**
23: **end for**
24:
$RC \leftarrow ~\{CR_{1}, CR_{2}, {\dots }, CR_{k}\}$
25: **for** $i \leftarrow $ 1 to $k$ **do**
26: **for** $t \leftarrow $ 1 to $n$ **do**
27:
$[M]_{it} \leftarrow ~RC$
28: **end for**
29: **end for**
30: **if** $CR$ is available **then**
31:
$[M]_{it} \leftarrow ~1$
32: **else**
33:
$[M]_{it} \leftarrow ~0$
34: **for** $t \leftarrow $ 1 to $n$ **do**
35:
$E_{RC} \leftarrow $ *External_Patient_Data* ( $R_{t}$)
36:
$CS \leftarrow ~Enc_{K_{RGA}}(E_{RC})$
37:
$A \leftarrow $ *CALL_* $\theta $*-TARNN* ( $R_{t}, f, cs$)
38: **for** $j \leftarrow $ 1 to $l$ **do**
39: **if** ( $A < R_{total}$) **then**
40: Map $P_{j} \rightarrow R$ based on $A$
41: Update $CR_{k}$ and output $B$ as *TRUE*
42: **else**
43: Output $B$ as *FALSE* and print *“Resources not available”*
44: **end if**
45: **end for**
46: **end for**
47: **end if**

## IV. The Proposed Forecasting Model

**FIGURE 4.**
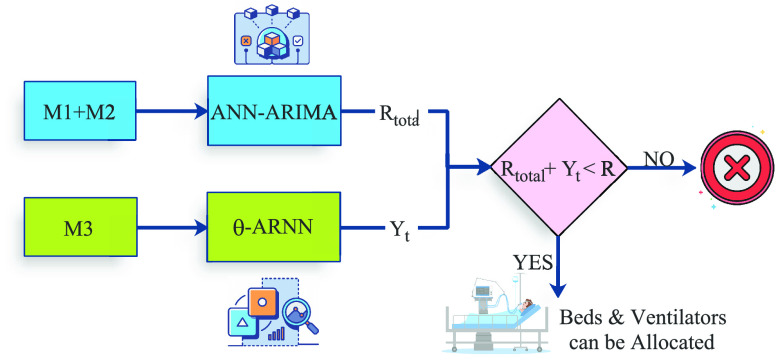
The forecasting scenario diagram.

This section presents the schematics of the prediction model. This forecasting is divided in two parts, one for the local captured data from $R_{n}$ based on $M_{1}$ and $M_{2}$, and another for external COVID-19 prediction taken from $M_{3}$. FIGURE 4 presents the schematics of the proposed forecasting scenario. For $M_{1}$ and $M_{2}$, we consider the estimation $R_{total}$, in terms of $CR=\{BA, V, OC\}$. We consider the collected data from $\{R_{1}, R_{2}, {\dots }, R_{n}\}$ and propose a hybrid approach that combines artificial neural networks (ANN), and auto-regressive integrated moving average (ARIMA) [26]. Although ARIMA models are considered suitable for time-series forecasting, we can apply different smoothing models due to their statistical nature. However, ARIMA in isolation has an inherent drawback of assumption of linear form, where the future values have a linear relationship with the current and past values. However, in the proposed scheme *ABV-CoViD*, the model has resource specification non-linear dependencies, which affect the consistency of modelling. Thus we, combine ANN with ARIMA to address the non-linearity of the model. With ANN, we capture the different non-linear relations in the underlying process, and thus a hybrid model is considered. However, no single model is considered superior in time-series forecasting, and it depends on the input patterns. Therefore, an optimal mix is selected.

For $M_{3}$ modelling, we consider the approach of the theta auto-regressive neural network model, commonly known as $\theta $-ARNN, to predict the rise in COVID-19 cases, that would address the requirements of $R_{total}$ for new $q$ patients. As COVID-19 patterns are non-stationary and non-linear [42]. ARNN forecasting has resulted in better forecasts than its linear counterparts for disease predictions. Moreover, the COVID-19 dataset has fewer data points for estimation, which might generate biased predictions, and neural nets can smooth the same [45]. Thus, $\theta $-ARNN allows an effective forecasting model with a fusion of linear and non-linear relationships with time-varying variance and complex auto-correlations. The model captures the linear patterns in the first phase, and ARNN captures the non-linear residues from the base $\theta $ model. $\theta $-ARNN, in general, has easy interpretability and produces accurate predictions. We present the details as follows.

### A. Estimation of $M_{1}$ AND $M_{2}$: The ANN-ARIMA Approach

**FIGURE 5.**
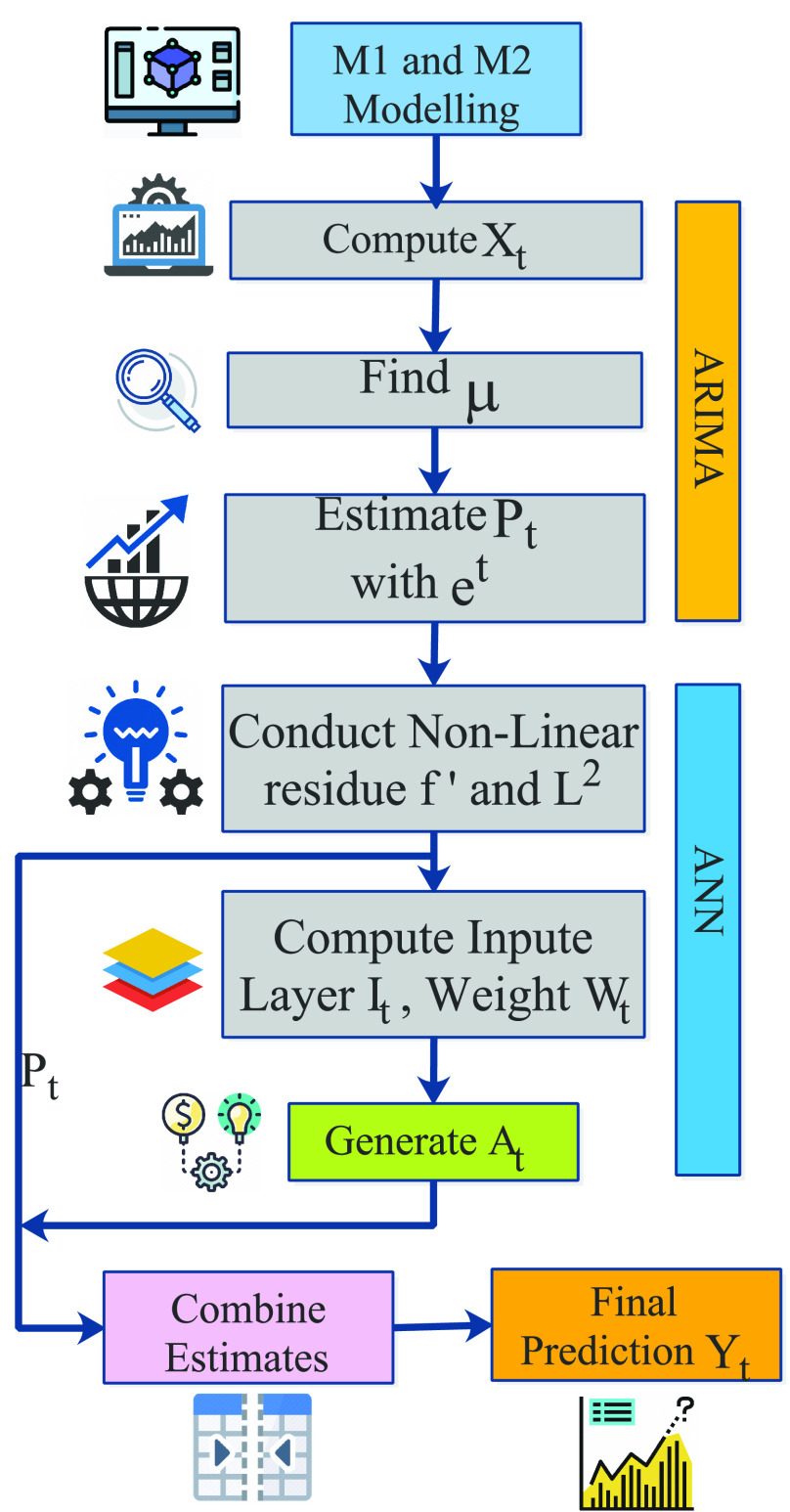
Flow diagram for $M_{1}$ and $M_{2}$ modelling based on ANN-ARIMA approach.

In ARIMA, we consider $ARIMA(p,d,q)$ model, where the future value of a variable is linearly dependent on past observations and random errors. FIGURE 5 presents the flow model of the modelling process, which applies the forecasting in two phases- the linear phase (ARIMA) and the non-linear phase (ANN). A time series with a mean $\mu $ is designed as follows.

\begin{equation*} \phi (W)\triangledown ^{l}(y_{t}-\mu)=\theta (W)a_{t}\tag{5}\end{equation*}

where $y_{t}$ and $a_{t}$ are considered the actual and random error at any time $t$, and $\phi (W)$ and $\theta (W)$ are polynomials of degree $m$ and $n$, which is presented as follows.

\begin{align*} \phi (W)=&\sum _{i=1}^{m}\psi _{i} W^{i} \\ \theta (W)=&1-\sum _{j=1}^{n}\theta _{j}W^{j}\tag{6}\end{align*}

We consider $\psi $ and $\theta _{j}$ as model parameters we like to investigate, with orders as $m$ and $n$. $\triangledown $ depends on the backward shift operator $W$ ( $1-W$), and $l$ denotes the linear differentiation operator. $a_{t}$ is assumed to be identically distributed with $\mu =0$, and variance of $\mu ^{2}$. We match the auto-correlation patterns with theoretical observations, as defined in Box-Jenkins [46] method. For non-linearity, we consider the ANN model dependent on three layers connected in an acyclic fashion. At any given time $t$, we have inputs $\{y_{t-1}, y_{t-2}, {\dots }, y_{t-f}\}$, which is connected to the output $y_{t}$ as follows.

\begin{equation*} y_{t} = a_{0}+\sum _{j=1}^{s}w_{j}g \left ({w_{0j}+\sum _{i=1}^{t}w_{i,j}y_{t-i}}\right)+e_{t}\tag{7}\end{equation*}

where $w_{i,j}$ represents model parameters with values $(1,2, {\dots },s)$, and $(1,2, {\dots },t)$ respectively, which are presented as connection parameters, $t$ is the input parameters as nodes, and $s$ is the hidden nodes. We use a sigmoid function with transfer function defined as follows.

\begin{equation*} \sigma (x)= \frac {1}{1+e^{-x}}\tag{8}\end{equation*}

Thus, the model maps $y_{t}$ to previous observations, denoted as $y(t)=f(y_{t-1}, y_{t-2}, {\dots }, y_{t-f}W)+e_{t}$, where $f(.)$ is the underlying function of ANN, and $W$ is the parameter vector. Equation 7 presents a one-step forward forecasting, and is able to approximate $f(.)$ with large $s$. The choice of $s$ depends on data, and input vector dimensions. In a time-series data, we present an auto-correlation structure as a function of linear and non-linear component. Thus, at any given time $t$, we have,

\begin{equation*} y_{t} = f(L_{t}, NL_{t})\tag{9}\end{equation*}

where $L_{t}$ and $NL_{t}$ denotes the linear and non-linear components respectively. initiate with $L_{t}$ as initial stage, presented as follows.

\begin{equation*} L_{t} = \hat {L_{t}}+ e_{t}\tag{10}\end{equation*}

where $\hat {L_{t}}$ represents the forecast value based on Equation (5), and $e_{t}$ presents the linear residue. At the second stage, we model the non-linear relationships through a multilayer perceptron (MLP) approach. We consider $\alpha ^{1}(t)$ and $\alpha ^{2}(t)$ as follows.

\begin{align*} \alpha ^{1}(t)=&f^{1} (e_{t-1}, e_{t-n}, {\dots }, e_{t-n}) \\ \alpha ^{2}(t)=&f^{2} (z_{t-1}, z_{t-2}, {\dots }, e_{t-m}) \\ \alpha _{t}=&f(\alpha ^{1}(t),\alpha ^{2}(t))\tag{11}\end{align*}

where $f^{1}$, $f^{2}$, $\alpha _{t}$ are non-linear functions based on the MLP network, and $n$ and $m$ are the model orders. The ANN forecast can be presented as follows.

\begin{equation*} y_{t} = f(\alpha ^{1}(t), \hat {L_{t}}, \alpha ^{2}(t))\tag{12}\end{equation*}

$f$ would be determined by ANN layers, with the trivial conditions $n_{1} \leq n$, and $m_{1} \leq m$. The nonlinear values might not be considered if the data has only linear dependencies. If the data is highly nonlinear, then the proposed *ANN-ARIMA* model captures the linear and nonlinear auto-correlations on data with higher accuracy over traditional time-series models. Algorithm 2 represents the details of the implementation of the ANN-ARIMA approach; this also represents the working models of the module- M1 and module- M2. Line number 2, 3, and 4 indicates the mean calculations of the $\mu $. Line numbers 6 and 7 denote the one-step forward forecasting using the variable $y_{t}$. The linear and nonlinear components were identified in lines numbers 16 to 26 and finally reached the equations of the forecast using $\alpha _{t}$ and $y_{t}$. Hence, the proposed ANN-ARIMA model captures linear and nonlinear autocorrelation with higher accuracy than the traditional time-series models.

Algorithm 2 **
$M1$ and $M2$ Modelling: The $ANN$- $ARIMA$ Approach**
Input: Hyperparameters $\{p,d,q\}$, $R_{total}$
Output: Forecast $y_{t}$
Initialization:
$i=1$, $j=1$, $f=1$, $\mathrm {z}=1$
1: **procedure** *ANN-ARIMA* ( $p,d,q,R_{total}$)
2:
$R \leftarrow ARIMA(p,d,q)$
3:
$R_{total} \leftarrow $ *Tune_Hyperparameters* ( $p,d,q$)
4:
$\mu \leftarrow \varnothing (W) {\nabla }^{l} (y_{t} - \mu) $
5: **if** ( $R_{e}= y_{t}$ and $A_{e} == a_{t}$) **then**
6: **for** $i \leftarrow 1$ to $m$ **do**
7:
$\varnothing (W) \leftarrow (\psi _{i}w^{i})$
8: **end for**
9: **for** $j \leftarrow 1$ to $n$ **do**
10:
$\theta (W) \leftarrow 1-\sum _{j=1}^{n}\theta _{j}W^{j}$
11: **end for**
12: **else**
13: **Print** *“Theoretical observations will not match with expected* $y_{t}$”
14: **end if**
15:
$t \leftarrow $ *current.timestamp*( $T$)
16:
$y_{t} \leftarrow f(L_{t}, NL_{t})$
17:
$L_{t} \leftarrow \hat {L_{t}}+e_{t}$
18: **for** $f \leftarrow 1$ to $n$ **do**
19: **for** $\mathrm {z} \leftarrow 1$ to $m$ **do**
20:
$\alpha ^{1}(t) \leftarrow f^{1} (e_{t-1}, e_{t-n}, {\dots }, e_{t-n})$
21:
$\alpha ^{2}(t) \leftarrow f^{2} (z_{t-1}, z_{t-2}, {\dots }, e_{t-m})$
22: **end for**
23: **end for**
24:
$\alpha _{t} \leftarrow f(\alpha ^{1}(t),\alpha ^{2}(t))$
25:
$y_{t} \leftarrow f(\alpha ^{1}(t), \hat {L_{t}}\alpha ^{2}(t)$
26: **end procedure**

In the next section, we present the $M3$ modelling through the $\theta -ARNN$ approach.

### B. Estimation of $M_{3}$: $\theta$-ARNN Approach

**FIGURE 6.**
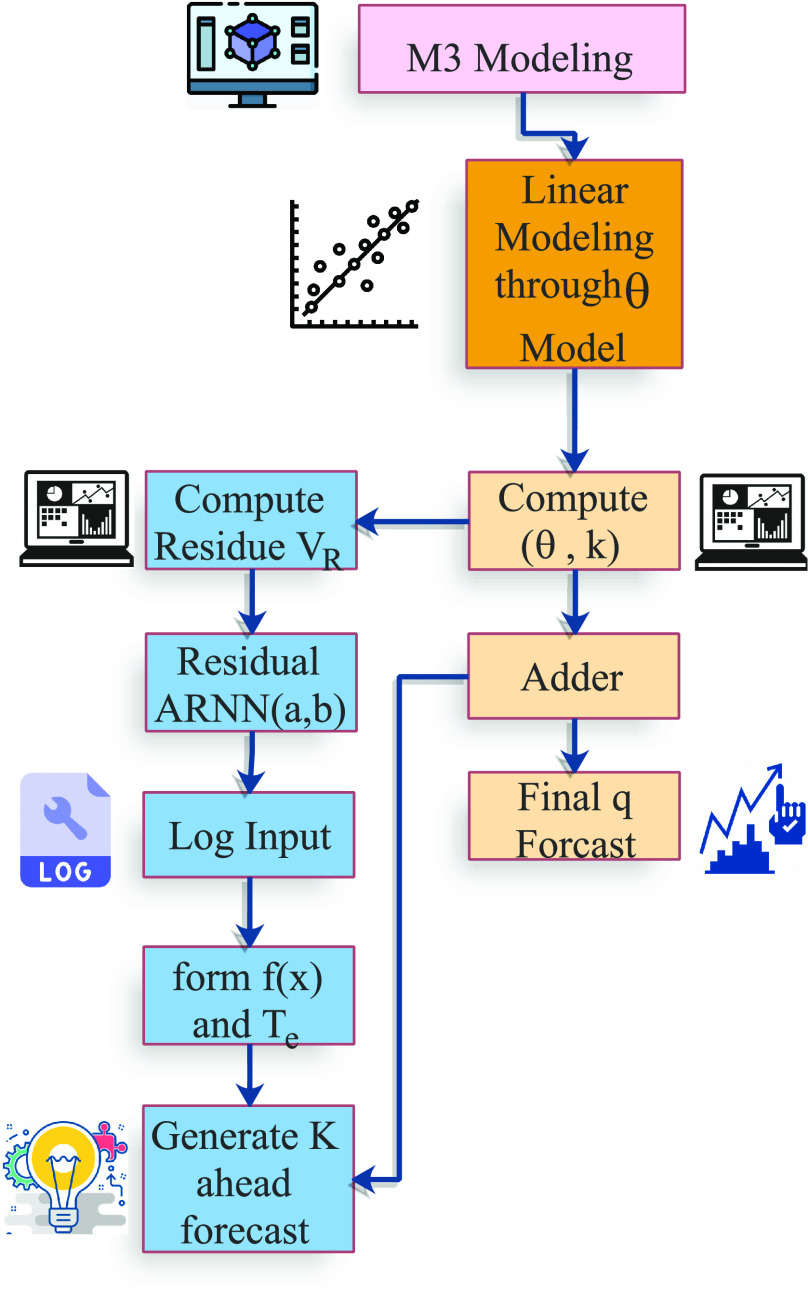
Flow diagram for $M_{3}$ modelling based on $\theta$-ARNN approach.

In this section, we discuss the $\theta $-ARNN to predict the rise in COVID-19 cases, which would quantify the requirements of $R_{total}$. FIGURE 6 presents the flow model of the $M_{3}$ modelling where we consider a $\theta $ model. It is a univariate time-series model. We assume that COVID-19 infected patients datasets are decomposed into more than two $\theta $ lines, and a forecasting model is applied to extrapolate the data. Intuitively, $\theta $ lines are curvatures on collected time-series data for $t_{n}$ persons who have their samples collected at $E_{RC}$. The $\theta $ coefficient is computed on the time series second-order differential, denoted as $T''(\theta)$, and is computed as follows.

\begin{equation*} T''(\theta) = \theta Y''(D_{t_{n}})\tag{13}\end{equation*}

where $D_{t_{n}}$ denotes the collected data of $n$ external persons, and $Y''(t) = Y_{t}-2Y_{t-1}+Y_{t-2}$ at given time instant $t \in [3], [n]$. $\theta $ denotes the slope transformation on original data with different variance values. Equation (13) has a solution represented as follows.

\begin{equation*} T(\theta) = a_{\theta } + b_{\theta } + \theta Y_{t}\tag{14}\end{equation*}

$a_{theta}$ and $b_{\theta }$ are trend coefficients and smooths the forecasting observations on weighted average values. The ARNN model is built on the standard ANN method and uses a specified lag value of time-series as input, with a fixed hidden neuron layer. In general, *ARNN* ( $f,g$) denotes $f$ lagged input with one hidden feedforward neural network, and $g$ hidden layer details. The network architecture $A^{*}$ is dependent on $\{f,g\}$ as follows.

\begin{equation*} A^{*} = \gamma _{0} + \sum _{q=1}^{r} w_{q} \epsilon (a_{q} + b'_{q}*)\tag{15}\end{equation*}

In this case, $\gamma _{0}, a_{q}, w_{q}$ denotes the connection weights, and $b_{q}$ denotes a $q$-dimensional weighted vector, which is supplied by the backpropogation model. Normally, $q = \frac {(f+1)}{2}$ is chosen in the ARNN method.

In the scheme, we consider the following definitions.

Definition 1 (Addicitve Forecast): In the addictive forecasting model, we consider an estimate $\hat {E}_{t}$ as sum of terms $E_{t}$ and $e_{t}$, denoted as $E_{t} + e_{t}$, where $E_{t}$ is the actual cases, and $e_{t}$ is the error or bias.

Definition 2 (Multiplicative Forecast): In the multiplicative forecasting model, we consider an estimate $\hat {E}_{t}$ as product of terms $E_{t}$ and $e_{t}$, denoted as $E_{t} \times e_{t}$.

The scheme applies the $\theta $ method initially to COVID-19 data to derive the linear components in the given dataset. Then, the generated residuals, and the addictive errors which are propagated by the $\theta $ method are again modelled using the nonlinear ARNN approach. The obtained forecasts are combined to determine the final forecast of the model. The mathematical model is defined as follows.

\begin{equation*} X_{t} = L_{t} + NL_{t}\tag{16}\end{equation*}

where $L_{t}$ and $NL_{t}$ denote the linear and non-linear parts in the $\theta $-ARNN approach. We derive the estimates $L_{t}$ and $NL_{t}$ form data. Based on $\hat {L}_{t}$, we consider the error residual $\epsilon _{t}$ at time instant $t$ for the $\theta $ model. Thus, we can write the residual as follows.

\begin{equation*} \epsilon _{t} = X_{t} - \hat {L}_{t}\tag{17}\end{equation*}

$\epsilon _{t}$ residuals are further designed through the ARNN model as follows.

\begin{equation*} \epsilon _{t} = f(\epsilon _{t-1}, \epsilon _{t-2}, {\dots }, \epsilon _{t-r}) + u_{t}\tag{18}\end{equation*}

where $f(.)$ is the non-linear function, and $u_{t}$ shows the sudden spike deviations in the plot. As depicted in Equation (15), the combined forecast is shown as follows

\begin{equation*} C_{t} = \hat {L}_{t} + X_{t}\tag{19}\end{equation*}

The combination is intuitive; $\theta $-ARNN remodels the residual correlation. Thus, it minimizes the error via remodeling, removes the COVID-19 time-series mishits, and smoothens out the noisy behavior of certain data points. Algorithm 3 mentions the M3 modeling and the $\theta $-ARNN mechanism. The input for this algorithm will be COVID-19 infected patients’ time series data, in terms of $R_{t}$, f, CS. As mentioned before, the $\theta $ component is computed on the time series second-order differential equation, and the notation for the same is $T{''}$ and is depicted in line number 5. The ARNN usage of the flagged input with one hidden feedback neural network results in $A^{*}$, and the same is mentioned in lines number 6 to 9. The errors residual will be identified using $\epsilon _{t}$. Finally, the model minimizes the error using remodeling, and this also removes the mishits and smoothens out the noisy behavior of the data points, which is depicted in line number 15.

Algorithm 3 **
$M3$ Modelling: The $\theta$-ARNN Approach**
Input: COVID-19 infected patients time-series data $R_{t}$, $f$, $CS$.
Output:
$\theta $-ARNN Forecast $X_{t}$, and the combined forecast indicator $C_{t}$
**Initialization:**
1: **procedure** $\theta $-*ARNN* ( $R_{t},f,CS$)
2: **for** $t \leftarrow $ 3 to $n$ **do**
3:
$T_{n} \leftarrow $ ( $R_{t}, f$)
4:
$R_{t} \leftarrow $ *Initialize_ $\theta $_lines* ( $T_{n}$)
5:
$T''(\theta) \leftarrow \theta Y''(D_{t_{n}})$
6: **if** $y''(t) \leftarrow \,\,Y_{t}-2Y_{t-1}+Y_{t-2}$ **then**
7:
$T(\theta) \leftarrow \,\,a_{\theta } + b_{\theta } + \theta Y_{t}$
8: **for** $q \leftarrow $ 1 to $r$ **do** $A^{*} \leftarrow \gamma _{0} + w_{q} \epsilon (a_{q} + b'_{q}*)$
9: **end for**
10: **end if**
11: **end for** $AF \leftarrow \,\,E_{t} + e_{t}\,\,MF \leftarrow \,\,E_{t} \times e_{t}\,\,X_{t} \leftarrow \,\,L_{t} + NL_{t}$
12: **for** $j \leftarrow $ 1 to $r$ **do**
13: Compute $\epsilon _{t}$
14: **end for**
15:
$C_{t} \leftarrow \,\,\hat {L}_{t} + X_{t}$
16: **end procedure**

## V. ABV-COVID: Performance Evaluation

In this section, we present the performance evaluation of the scheme in terms of $M_{1}$ and $M_{2}$ forecasting via the ANN-ARIMA approach and $M_{3}$ forecasting using the $\theta $-ARNN approach. For forecasting models, we have considered the COVID-19 healthcare dataset [47]. The data is updated daily, showcasing cumulative deaths, daily deaths, vaccine coverage, hospital resources use, infection and testing, and other related information. We have considered 3 regions, which is represented as $R1, R2, R3$, where $R1$ (Region-1 for Illinois), $R2$ (Region-2 for Michigan), and $R3$ (Region-3 for Indiana). The data is considered for a time period from Jan 2021 to May 2022, and we have presented our predictions on ABV resources based on COVID-19 cases (till August 2022). TABLE 4 presents the details of the total population, the confirmed cases, and the total deaths for the three regions. The details of the simulation parameters are presented in the next subsection.

**TABLE 4:** Comparative Population Analysis

Region	Population	Total +ve	Deaths
$R1$	12671821	3169314	37956
$R2$	9986857	2444891	36064
$R3$	6732219	1707085	23614

### A. Simulation Parameters

This section describes the various simulation parameters used to carry out the experimental work. TABLE 5 indicates about the various parameters. For the given regions $\{R_{1}, R_{2}, R_{3}\}$, we have considered modelling through $ARIMA(p,d,q)$ using the *forecast* package in $R$ software. In the ANN modelling, $N^{8-3-1}$ is designed on the *MATLAB7* package, where the best fitted ANN model is selected using the pruning algorithm. The model consists of 8 inputs, 3 hidden layers, and 1 output neuron. For the $\theta $-TARNN, we have used the *thetaf* function, where the non-linear modelling uses the *caret* package. After fitting the $\theta $-model, we consider a 10 days ahead window and use the Box-Cox transformation with $\lambda = 0$, and the $ARNN(12,6)$. The next subsection discusses the performance metrics considered in the evaluation.

**TABLE 5:** Simulation Parameter Table

Parameters	Values
No of regions $R_{n}$	3
Hospitals $H_{k}$ (per $R_{n}$)	2
Patients $P_{l}$ (per $H_{k}$)	9585
$\{T_{l}, O_{l}\}$	2
ARIMA(p,d,q)	ARIMA(1,0,12)
Total Observations $N$	57,510
Stepsize	0.03
Random Model	Uniform in [−1,1]
Non-linear $M_{2}$	$N^{(8-3-1)}$
$\theta $- ARNN	10 days ahead
ARNN(p,k)	$\lambda $= 0 and ARNN(12,6)

### B. Performance Metrics

For metrics selection, we have considered the MAE, RMSE, DS, and MAPE for the given set of COVID-19 datasets. The details of the metrics are presented as follows.

#### 1) Mean Absolute Error

Statistically, MAE is defined as the error measurement between paired observations, where the plots present the information of predicted versus observed. In time-series forecasting, it is computed as an average of forecast error values (positive), and thus is termed as absolute, as we force the values to be only positive. Mathematically, it is represented as follows.

\begin{equation*} MAE=1/n \sum _{i=1} ^ {n} |y_{i} - \hat {y_{i}}|\tag{20}\end{equation*}

where $y_{i}$ is the actual value, and $\hat {y_{i}}$ is the predicted value, and $n$ denotes the number of observations. Thus, MAE is not directional, as it only measures the error magnitudes of time-series data.

#### 2) Root Mean Squared Error

RMSE is also an absolute error measurement that computes the squares of deviations. This does not allow positive and negative deviations to cancel themselves. In time series, it indicates an absolute fit of the model to data and measures the closeness of model-predicted values to observed data points. It is the standard deviation of the variance, and lower values of RMSE indicate a better fit for the model, as it measures the accuracy of model prediction to actual response. Mathematically, it is presented as follows.

\begin{equation*} RMSE= \sqrt {1/n \sum _{i=1} ^ {n} (y_{i} - \hat {y}_{i})^{2}}\tag{21}\end{equation*}

where $y_{i}$ is the actual value, and $\hat {y_{i}}$ is the predicted value, and $n$ denotes the number of observations. It is also a non-directional measurement.

#### 3) Mean Absolute Performance Error

MAPE, better known as mean absolute percentage deviation (MAPD), is a relative measurement that uses absolute values like MAE and allows for comparing the forecast accuracy in time series. MAPE is mainly used for forecast error measurement, as the probability units are scaled to percentage values. Similar to MAE, MAPE is accurate in case data has no extremes. The formulation of MAPE is expressed as follows.

\begin{equation*} MAPE=(1/FC) \sum _{t=1}^{FC}|\hat {X_{t}}-X_{t}/X_{t}| \times 100\tag{22}\end{equation*}

where $FC$ denotes the total number of forecasts in the testing set, $X_{t}$ is the original value of the provided time series at time given time $t$, and $\hat {X_{t}}$ is the predicted value.

#### 4) Directional Statistics

DS deals with unit vectors in $R^{n}$, and presents direction-based observations. It is mainly used in mainstream statistical methodology. The data which is examined is represented as Riemannian manifolds in form of unit circle, sphere, torus, and their extensions in a variety of scenarios. It is supported by novel applications in fields as diverse as astronomy, pharmaceutics, genetic factors, neuroscience, aerospace engineering, acoustic performance, image processing, text analysis, ecologic metrics, and deep learning operations.

The observation of the direction is in the plane named as $\mathbb {R}^{2}$. As an example, the wind direction can be represented by an angle $\theta $, and typically this will be in the range of $[0, 2\pi$) or $[-\pi, pi$) measured in a specific direction from a specific origin. The same can be represented as a unit vector $x= (Cos\theta, Sin\theta)'$ for which the value is $||x||= \sqrt {X'X}=1$. The circumference of the unit circle $C1$ provides the natural support for such mentioned directions. Thus, it is referred to as circular statistics. The term circular data is also used to make a distinction from the data that has the real line $\mathbb {R}$ (or a small subset of this) as its support and is referred to as linear data. The data on these various manifolds and their generalizations, such as the unit $d$-dimension sphere, and its representation is in the form of $\mathbb {S}^{d}$ and the d-torus is denoted as $\mathbb {T}^{d} = (S^{1})^{d}, d\leq 1$. The outcome of this result may be observed and analyzed for regressions, spatial, time series, or Spatio-temporal data. Data that do not originally have directions but may be expressed on, or transitioned to, one of the previously mentioned manifolds can also be used with DS.

Hence, the DS is more suitable for business applications because investors are interested in the market trend. As a result, DS is used to compare competing models’ directional forecasting accuracy and is defined as follows.

\begin{equation*} DS=(1/FC) \sum _{t=1}^{FC} D_{t} \times 100\tag{23}\end{equation*}

Here, the $D_{t}=1$, if $(X_{t+1} -X_{t}) \times (\hat {X_{t+1}} - X_{t}) \geq 0$, or else the value of the $D_{t}=0$. The $FC$ corresponds to the number of total testing set of forecasts. Hence, the lower the value of the MAPE, the higher is the forecasting accuracy with respect to the higher value of the $DS$. This leads to the effective accuracy in the result.

### C. Performance Metrics Evaluation

In this subsection, we present the performance metrics evaluation for our proposed scheme. As indicated above, we consider three regions $R1$, $R2$, and $R3$ for our evaluation, and we compute the value of MAE, RMSE, MAPE, and DS for five models- ARIMA, Theta, ARNN, the proposed ANN-ARIMA, and the proposed $\theta $-ARNN (TARNN) method. The details of the evaluation are presented as follows.

TABLE 6 showcase the results obtained through the RMSE of the regions $R1$, $R2$, and $R3$. The RMSE has been evaluated using different schemes like ARIMA, Theta, ARNN, ANN-ARIMA, and $\theta $-ARNN (TARNN). The evaluations show that the $\theta $-ARNN received the lowest RMSE value of 641.6701 compared with the aforementioned schemes. Similarly, for the region $R2$, the lowest value of the RMSE is calculated for the scheme *ANN-ARIMA* and the value is 134.7698. In the case of $R3$ the minimum RMSE is received for the scheme $\theta $-ARNN and the value is 192.0985.

**TABLE 6:** Performance Based on the Value of RMSE

Region	ARIMA	Theta	ARNN	ANN-ARIMA	$\theta $-ARNN (TARNN)
$R1$	3218.3912	4103.4500	1289.6700	2956.8977	641.6701
$R2$	4367.8719	287.7890	317.7657	134.7698	165.7809
$R3$	701.4500	213.7600	789.6578	219.9800	192.0985

Similarly, TABLE 7 presents the MAE values. For the region $R1$, the lowest value is calculated for the scheme TARNN and the value is 321.4332. The region $R2$ lowest MAE is calculated using the scheme *ANN-ARIMA* and its value is 65.7892. The $R2$ minimum error for the MAE is achieved as 108.5432 using the scheme TARNN, where $y_{i}$ is the original rate, and $\hat {y_{i}}$ is denoted as predicted value. The number of data point is depicted by the variable $n$.

**TABLE 7:** Performance Based on the Value of MAE

Region	ARIMA	Theta	ARNN	ANN-ARIMA	$\theta $-ARNN (TARNN)
$R1$	2190.5672	2256.6734	1065.2134	2221.4562	321.4332
$R2$	2344.7866	3876.5644	176.6671	65.7892	81.6778
$R3$	321.8766	321.7806	321.7654	123.6532	108.5432

TABLE 8 presents the MAPE values for regions $R1$, $R2$, and $R3$ respectively. We evaluate MAPE for ARIMA, Theta, ARNN, ANN-ARIMA, and $\theta $-ARNN. For $R1$, the evaluation results shows that ARNN achieves the lowest MAPE value of 1378.4184. For $R2$, ANN-ARIMA performs the best with 169.8973, and for $R3~\theta $-ARNN gives the lowest MAPE value of 328.7865.

**TABLE 8:** Performance Based on the Value of MAPE

Region	ARIMA	Theta	ARNN	ANN-ARIMA	$\theta$-ARNN (TARNN)
$R1$	4482.8723	5892.2479	1378.4184	3224.3256	826.2871
$R2$	5944.7898	3182.8129	4202.8223	169.8973	289.6799
$R3$	829.8145	512.8856	868.1246	334.8984	328.7865

Next, we evaluate the performance based on DS. It is important metric which allows the healthcare stakeholders to assess the BV requirements, based on the COVID-19 forecasts for the regions. TABLE 9 presents the results. For $R1$, the proposed $\theta $-ARNN has the best accuracy with 87.83%. For $R2$, the ANN-ARIMA model gives an accuracy of 93.87%, and for $R3~\theta $-ARNN again performs significantly better than other schemes, with a value of 94.87%. FIGURE 8 presents the results.

**TABLE 9:** Performance Based on the Value of DS (in %)

Region	ARIMA	Theta	ARNN	ANN-ARIMA	$\theta$-ARNN (TARNN)
$R1$	62.45	72.29	58.65	63.84	87.93
$R2$	68.93	63.67	53.89	97.64	93.87
$R3$	64.32	54.87	88.59	91.89	94.76

**FIGURE 7.**
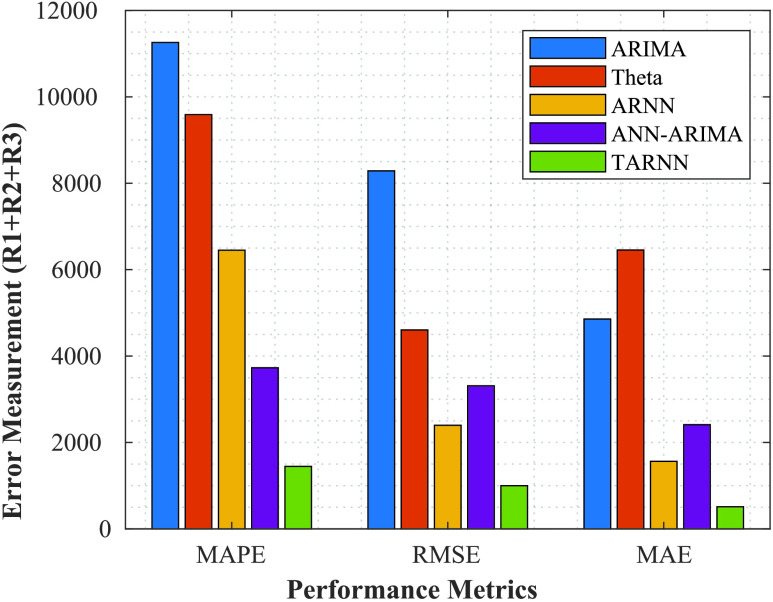
Forecasting accuracy measured of regions $R1$, $R2$, $R3$ combined.

**FIGURE 8.**
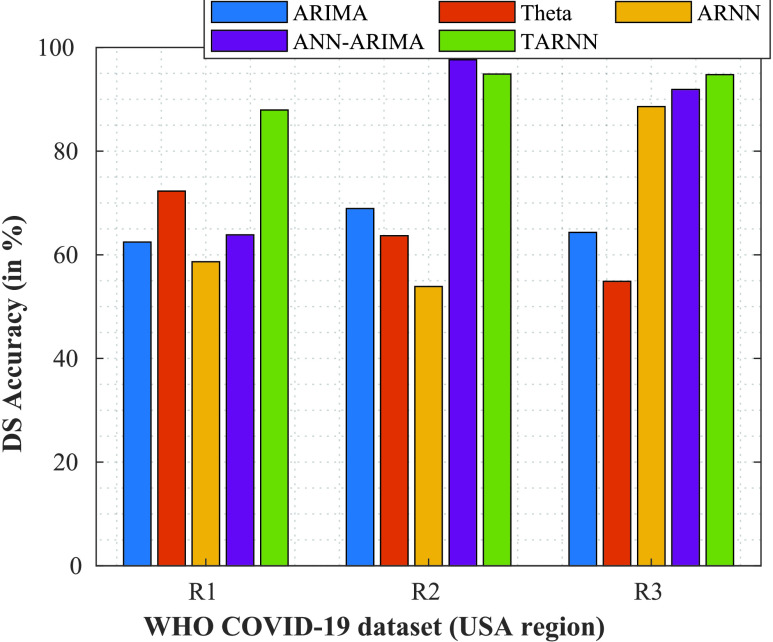
DS Plots of different models for regions $R1$, $R2$, and $R3$.

Next, we evaluate the forecasting accuracy of the models for the three regions combined $R1 + R2 + R3$. We have considered three metrics- MAPE, RMSE, and MAE for our proposed models ARIMA, Theta, ARNN, ANN-ARIMA, and $\theta $-ARNN (TARNN). FIGURE 7 depicts the forecasting accuracy results. The results show that the MAE is providing better results as compared to the other performance indicators/metrics, such as RMSE and MAPE. Also, in all cases, the ensemble model ANN-ARIMA, and TARNN have a good performance in all indicators.

For singular models like ANN, ARIMA, and Theta, the DS performance is not satisfactory, owing to the complex nature of COVID-19 nonlinearity in the dataset. However, when we consider the ensemble models, ANN-ARIMA and $\theta $-ARNN, we get effective results. For the same, the RMSE, MAE, and MAPE values have a higher accuracy (minimum values), which validates our claim that ensemble models have better performance. The reason is trivial, as we capture both linear and non-linear dependencies for short-term and long-term forecasting. Next, we present the details of the performance evaluation of the proposed scheme. We discuss the hardware results, and then present the simulation results in detail.

### D. Hardware Setup

An experimental setup has been carried out using the *OpenStack* private Cloud consisting of three Virtual Machines (VMs) that form the forecasting analytics. $M_{1}$ and $M_{2}$ modelling is done on the first VM, and the $M_{3}$ modelling is carried out in the next two VMs. The hardware details where the VMs are installed consist of *PDL380* $10^{th}$ generation of rackmount server, MS Windows server standard core 2019 sngl olp 16 lic-ae, *Intel XEON* silver 4110, 128 GN (32GB*4) DDR4-2666 MHz, 2.4 TB SAS 12G 10k SFF HDD, and HPE Smart Array 8161-a SR 10th Gen CNTRLR.

### E. Simulation Results

The experimental evaluation for all the datasets mentioned here has been performed on the time series forecasting tools, such as Classical, Advanced, and Hybrid models. ARIMA is an example of the Classical approach; the ARNN belongs to the Advanced, and the $\theta $-ARNN is from the Hybrid approach.

**FIGURE 9.**
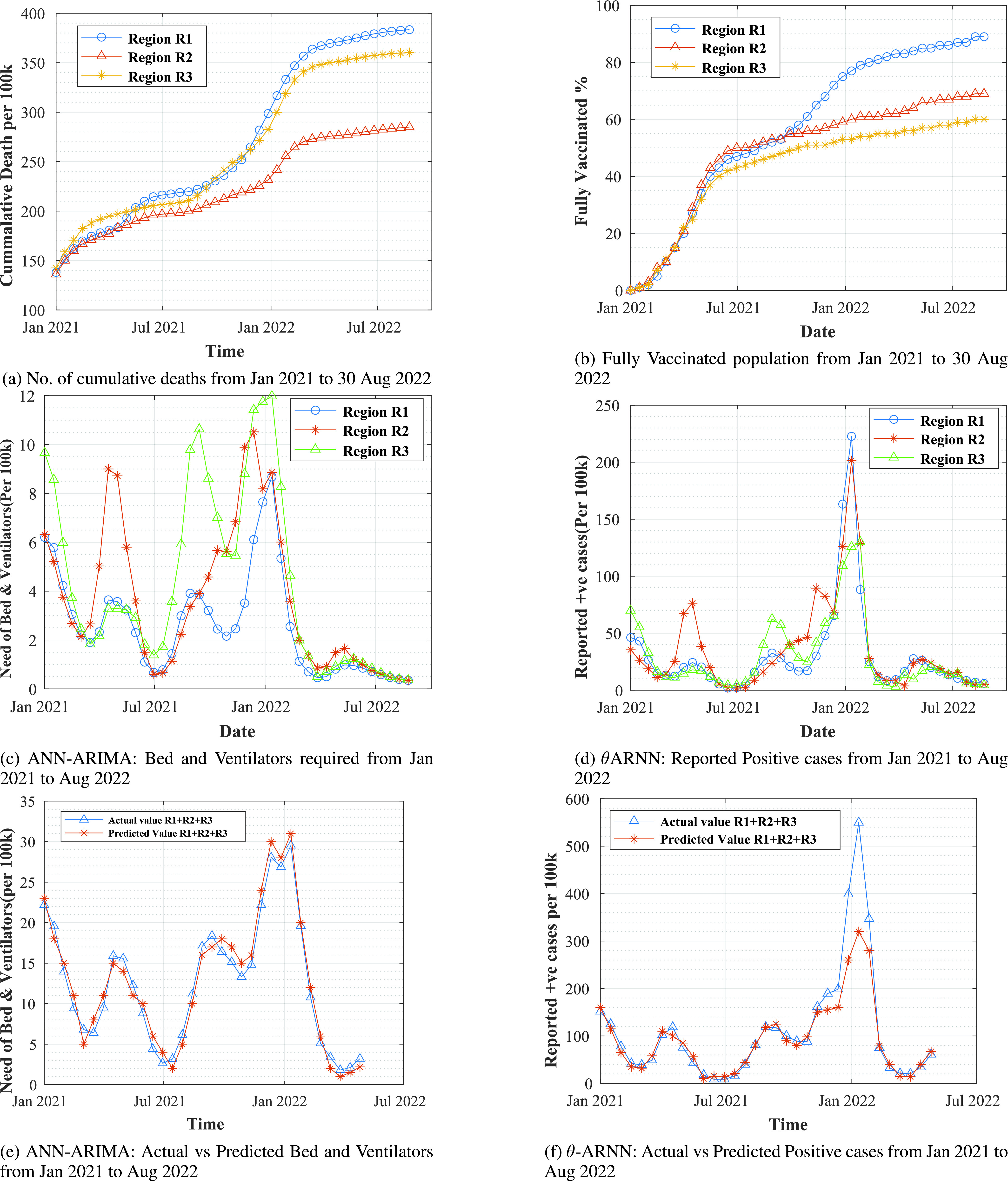
Simulation and prediction results of the *ABV-CoViD* scheme.

FIGURE 9a presents the cumulative deaths of patients admitted in hospital from January-2021 to May-2022 for region $R1$, $R2$ and $R3$. The analysis is done on region $R1$ with a target population of $\approx ~12.6$ million, $R2$ with a target population of $\approx 9.9$ million, and $R3$ with a target population of $\approx 6.7$ million. In region $R1$, the death rate of January 2021 shows $\approx 138$ deaths from $100k$ positive reported samples, while the death rate in May 2022 shows $\approx 371$ deaths from $100k$ reported samples, i.e., a drastic rise of 167.213% from the last year January month. Similarly, for the $R2$ region, there is a rise of 96.42%, and $R3$ has $\approx 150$% rise in deaths per $100k$ population. This shows an alarming situation, and the major cause of such deaths includes improper usage of critical resources.

FIGURE 9b represents the percentage of fully vaccinated population in each region. In January 2021 region $R1$, $R2$, and $R3$ has nearly $\approx 1$% population got fully vaccinated, while in January 2022 75%, 59.5%, and 52% respectively that shows an improvement of 74%, 58%, and 51% growth per $100k$ population for vaccine distribution and administration. Such vaccination programs develop immunity that helps fight against COVID-19 variants and reduce the chances of being in critical conditions where they require critical care units.

FIGURE 9c represents the use of BV per $100k$ reported cases in the regions $R1$, $R2$, and $R3$ from January 2021 to April 2022, and rest of the prediction till August 2022 is computed through *ANN-ARIMA* model, that takes hyper-parameters $p$, $d$, $q$, and $R_{total}$ as an input to the algorithm 2. *ANN-ARIMA* predicts the need for BV in each region based on the past requirements, recovered COVID-19 positive patients, and current positivity of the patients calculated by the $\theta -ARNN$ model. The figure clearly shows the ups and downs of the requirement of critical resources. If such behaviour is predictable, this prediction helps the network chain hospitals or regions get the ABV resources and creates the possibility of exchanging such resources among the network hospitals and regions.

FIGURE 9d represents the reported positive cases per 100k population from January 2021 to April 2022 of different regions and the rest of the prediction is computed through $\theta -ARNN$ algorithm 3. The model takes covid-19 infected patients time-series data $R_{t}$, $f$ and CS, with this input $\theta -ARNN$ compute $C_{t}$, that predict the positivity rate from May 2022 to August 2022 based on the current vaccination of different regions.

FIGURE 9e validates the prediction of actual used BV used against the predicted number of BV over the time series data from January 2021 to April 2022 combined for three regions ( $R1 + R2 + R3$). To compute this, we consider the reported COVID-19 positive case over per $100k$ population and the count of recovered cases. Based on the predicted values, accuracy of 98.876% is achieved. This future prediction helps the different regions to exchange hospital resources.

FIGURE 9f validates the prediction of actual COVID-19 positive cases reported versus predicted number of number of positive cases over the time series data from January 2021 to April 2022 of combined three regions ( $R1 + R2 + R3$). To calculate this, a fully vaccinated population over per $100k$ and recovered cases are considered and based on the predicted value, and we reached an accuracy of 98.752%.

### F. Key Findings

We summarize the key findings as follows.

1) We showcased the performance metrics evaluation for the WHO COVID-19 dataset for the three regions in the USA and computed values of RMSE, MAE, MAPE, and DS. For RMSE, our proposed ANN-ARIMA and $\theta $-ARNN model outperformed the singular ARIMA, Theta, and ARNN methods. The same trend is obtained in MAE and MAPE. However, for regions $R2$ and $R3$, ANN-ARIMA and TARNN showed higher accuracy.
2) Based on RMSE, MAE, and MAPE, we get higher accuracy in DS for ANN-ARIMA and TARNN. As both models capture the long-term dependencies of the data, we obtain better accuracy. The ensemble ANN-ARIMA performed better than $\theta $-ARNN for the $R2$ region. For the $R3$ region, $\theta $-ARNN outperformed the remaining models. Thus, a certain direction is predictable from this analysis. Thus, this validates our hypothesis about the inclusion of ensemble models like ANN-ARIMA and $\theta $-ARNN to capture both the short-term and long-term dependencies.
3) In simulation results, we considered the time window from January 2021 to August 2022. We computed the death rates in the regions. For every 100k positive sample, the trend shows an increase in death rates from January 2021. We considered the vaccination scenario in the same duration, and it shows an improvement, showing an increase in vaccination dosage. Thus, it means that people have turned careless towards the COVID-19 norms, and are not following the sanitization and distance measures seriously. The death rate has increased due to the new mutation in COVID-19 genetic sequences, and the Omicron XE variant.
4) Next, we measured our BV resource measurement, where we achieve a significant accuracy of 98.876% in the prediction of COVID-19 positive cases in time. Thus, it helps our healthcare setups to prepare in advance with the required BV units at hospitals, and prepare for timely actions.

## VI. Limitations and Future Strategies

In this section, we discuss the potential limitations in our study and present the possible directions. The results presented an inherent limitation in the forecasting models. In all cases, the ensemble methods ANN-ARIMA and TARNN performed better than singular models like ARIMA, Theta, and ARNN. However, for different regions, the performance indicators did not specify a unified trend. For regions $R1$ and $R3$, $\theta $-ARNN performed better, but for region $R2$, ANN-ARIMA performed better. Thus, formulating the exact reasons as to how certain model performs better than other is not straightforward [48]. COVID-19 data is highly complex and depends on varied factors like geographical conditions, the population of the considered regions, and the healthcare innovation in the considered regions. Variations in COVID-19 testing measures and local data collection play an important role in data quality. Thus, pinpointing a single model to cater for all metrics is difficult. Moreover, the data is highly dependent on sudden outbreaks, and involve subjectivity to evaluate the concerned parameters. Most models have higher accuracy for short-term and medium-term forecasting but fail to give significant results in long-term forecasts.

Due to this, it limits our predicted projections on the required quantities of BV resources at hospitals, as a sudden outbreak changes the forecasting dynamics. Also, the ABV modelling is suitably fit for short regions but does not provide good predictions for densely populated regions. Lack of training and historical data also limits researchers in accurate modelling and forecasting. At present, the availability of COVID-19 surveillance data is updated on weekly basis on different public platforms. Recent studies have suggested the modelling of COVID-19 on 2020 data, which is older and does not capture the recent COVID-19 third wave information. This limits its usage for accurate forecasting analysis. It would be thus interesting to remodel the forecast with the latest available COVID-19 datasets, which would provide higher accuracy. Also, time-series models have limitations based on national regulations (in terms of exogenous factors like correct data disclosure of health setups, vaccine availability, and BV units present at each hospital [49]). In real scenarios, these factors play a dominant role in forecasting analysis. With updated data, more analysis in terms of vaccine velocity, hospital capacity, and lockdown constraints can be effectively formulated. This would improve our long-term forecasting accuracy of the models.

As future strategies of the work, different AI techniques such as fuzzy-based analysis, DL models, and genetic algorithms can be integrated with forecasting models. In general, ensemble methods perform better, as they help in reducing the error rates and take into account the multivariate time series conditions. However, still, the interpretation of the prediction results needs to be justified. Thus, another future scope is to use explainable AI models like local interpretable model agnostic explanations (LIME), and Shapley additive explanations (SHAP) can be integrated with our forecasting models to signify the weightage of the considered parameters [50], [51]. Another future direction is the usage of the federated transfer learning approach in forecasting analysis. As collected data is highly sensitive and heterogeneous, it can be trained locally at hospital intrinsic setups, and the results (weights, interconnection, and lower layers representational value) can be transferred from one time-series model to another [52]. This would greatly minimize the biases in the model prediction, which improves the forecasting model accuracy.

## VII. Conclusion

Recently, the world has witnessed the detrimental effects of new variants of COVID-19. COVID-19 third wave has taken a serious toll on the local hospital setups, and thus every nation must identify and orchestrate effective healthcare policies and timely dissemination of critical resource management. We present a scheme, *ABV-CoViD*, that forms an ensemble forecasting of the BV resource availability based on the future requirements (which are predicted through COVID-19 forecasting). We considered two approaches for forecasting. The *ANN-ARIMA* model access the ABV requirements inside the local hospital setups in every region. To capture the future requirements and external COVID-19 positive cases, we proposed a $\theta $-ARNN model that predicts the rise in COVID-19 cases. We model our formulation based on different performance metrics like MAPE, RMSE, MAE, and DS. The results demonstrated that ensemble ANN-ARIMA and $\theta $-ARNN performed significantly well in all cases than singular ARIMA, Theta, and ARNN models, due to effective conditioning of the model complexity and non-linearity. This makes our scheme effective in short and long-term forecasts, which helps in formulating decisions of timely ABV resource management policy at a regional scale. As a use-case, we have considered the deployment of the scheme in three regions in the USA and considered metrics like cumulative deaths, vaccinations, BV requirements, and reported COVID-19 cases by both *ANN-ARIMA*, and $\theta $-ARNN method. We report an accuracy of 98.752% is reported for all three regions, which justifies an effective fit of BV mapping with COVID-19 positive case predictions.

As confirmed COVID-19 cases are spatially heterogeneous and time-varying, the model has an inherent limitation of best fit for $10-20$ days period but might not be accurate for larger time windows. Thus, as a future scope, the authors would extend the work by considering the Gaussian distribution nature that can capture higher accuracy for large time windows.
